# Mechanism of arbutin in metabolic dysfunction-associated fatty liver disease based on multi-omics research

**DOI:** 10.1186/s40643-026-01032-5

**Published:** 2026-03-25

**Authors:** Dapeng Yin, Jiacheng Cheng, Huili Cao, Xiaojuan Wang, Junhua He, Yikun Zhu, Jin Li

**Affiliations:** 1 Division of Endocrinology, Department of Medicine, The Second Hospital of Shanxi Medical University, 382 Wuyi Road, Xinghualing District, Taiyuan, Shanxi China; 2 Division of Cardiology, Department of Medicine, The Second Hospital of Shanxi Medical University, Taiyuan, Shanxi China

**Keywords:** MAFLD, Arbutin, Apoptosis, Gut microbiota, Fecal metabolites

## Abstract

**Introduction:**

Gut microbiota regulation is a key strategy for treating metabolic dysfunction-associated fatty liver disease (MAFLD). Arbutin (ARB) is a natural hydroquinone active agent with anti-inflammatory and antioxidant effects, as well as regulatory effects on the gut microbiota. However, its therapeutic effect on MAFLD and the responsible mechanisms remain unclear.

**Objectives:**

This study explored the therapeutic effect and mechanisms of ARB in MAFLD treatment.

**Methods:**

High-fat diet (HFD)-fed mice served as the in vivo MAFLD model, and ARB treatment was given simultaneously. The extent of liver injury was assessed through histopathological staining. AML12 cells treated with free fatty acids served as the in vitro model. The effects of ARB were evaluated via oil red O staining and biochemical assays. Subsequently, we utilized bioinformatics techniques to predict the potential mechanisms and targets of ARB. The expression of liver apoptosis-related genes was detected using molecular biology techniques. Alterations in the gut microbiota were analyzed by 16S rRNA sequencing. Ultrahigh-performance liquid chromatography–high-resolution mass spectrometry was used to analyze the changes in fecal metabolite levels.

**Results:**

ARB treatment effectively improved liver injury in mice with MAFLD. Its mechanism was associated with anti-apoptotic effects mediated by signal transducer and activator of transcription 3. Meanwhile, ARB effectively reversed gut microbiota imbalance in mice with MAFLD and altered the composition of gut microbes and fecal metabolites.

**Conclusion:**

ARB displayed potential effects in alleviating the pathology of MAFLD, exerting anti-apoptotic actions, and restoring the gut microbiota balance.

**Graphical Abstract:**

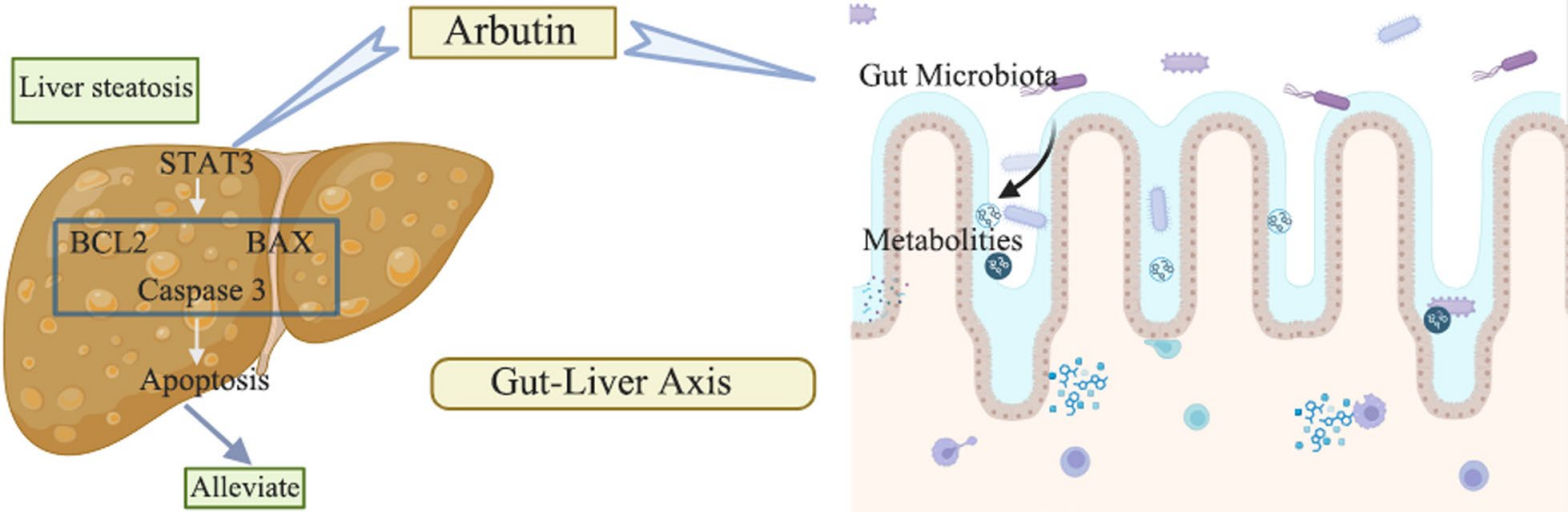

**Supplementary Information:**

The online version contains supplementary material available at 10.1186/s40643-026-01032-5.

## Introduction

Metabolic dysfunction-associated fatty liver disease (MAFLD), formerly known as nonalcoholic fatty liver disease (NAFLD), is the most common chronic liver disease in Western countries, and it is expected to become the most common reason for liver transplantation by 2030 (Byrne and Targher 2015). MAFLD is a complex metabolic disease that is often accompanied by systemic metabolic disorders such as obesity, insulin resistance, dyslipidemia, chronic low-grade inflammation, and gut microbiota imbalance (Rong et al. 2022). MAFLD encompasses a broad spectrum of liver pathologies, ranging from simple steatosis to steatohepatitis, fibrosis, and cirrhosis. Excessive fat accumulation in hepatocytes is the initial factor for the development of MAFLD. Despite numerous studies exploring potential pharmacological treatments, there is currently no consensus on the optimal therapy for MAFLD.

Lipid overload in hepatocytes generates lipotoxicity, which affects cell behavior through multiple mechanisms, including death receptor activation, endoplasmic reticulum stress, oxidative stress, and inflammation (Marra and Svegliati-Baroni 2018). Abnormal accumulation of specific lipid metabolism intermediate products, such as triglycerides (TG), total cholesterol (TC), free fatty acids (FFA), hemolytic lecithin, and ceramides, exceeds the normal compensatory capacity of hepatocytes, resulting in direct cellular damage (Jiang et al. 2025). These lipid metabolites can trigger oxidative stress, reactive oxygen species release, and Janus kinase (JAK)–signal transducer and activator of transcription (STAT) pathway activation (Shu et al. 2020). The JAK–STAT pathway influences the expression of pro-apoptotic (e.g., BAX, BAK) and anti-apoptotic proteins (e.g., BCL2, Bcl-xl), subsequently leading to hepatocyte apoptosis and irreversible damage (Mota et al. 2016). Therefore, regulating lipotoxic-induced apoptosis represents a potential treatment approach for MAFLD.

The occurrence of MAFLD is closely related to the influence of the gut microbiota on metabolism. Through the gut microbiota, microbial metabolites achieve bidirectional communication with the liver through the liver–gut axis (Pabst et al. 2023). Changes in microbial abundance, microbiota malnutrition, and changes in metabolite levels can promote intestinal barrier permeability disorders. This allows bacteria, endotoxins, and harmful metabolites to penetrate the intestinal barrier and translocate into the liver, affecting host immunity and inducing a low-grade systemic inflammatory state (Di Vincenzo et al. 2024). In addition, certain gut microbes directly regulate the production of short-chain fatty acids (SCFAs), exerting beneficial effects on hepatic lipid metabolism (den Besten et al. 2013). Metabolites of the gut microbiota, such as bile acids, can enter the liver through the portal vein and act as signaling molecules to interfere with hepatocyte metabolism (Qi et al. 2025). Accumulating evidence indicates that the gut microbiota plays a key role in metabolic diseases such as MAFLD. Therefore, targeting the gut microbiota to modulate host metabolic balance is a promising therapeutic approach.

Arbutin (ARB), a β-d-glucoside derivative of hydroquinone, is a bioactive substance isolated from the leaves of Ursa plants in the Ericaceae and Saxifragaceae families (Zhou et al. 2019). ARB is used in the pharmaceutical industry because of its antioxidant, anti-inflammatory, anti-fibrotic, anti-bacterial, and whitening effects (Liu et al. 2024). Recent studies have found that ARB can regulate host metabolism and the gut microbiota. Several studies have demonstrated that ARB treatment can reduce serum TC, TG, and low-density lipoprotein cholesterol (LDL-C) levels in mice (Jiang et al. 2023; Ma et al. 2022). The beneficial effects of ARB are achieved through improvements in intestinal development, gut microbiota regulation, and reductions in white adipocyte differentiation (Bedi et al. 2020; Ma et al. 2022). Furthermore, ARB can alleviate insulin resistance and improve blood glucose level in mice with type 2 diabetes (Madić et al. 2021). Based on the effects of ARB on metabolic regulation, it might represent a potential treatment for MAFLD. This study conducted target prediction through bioinformatics analysis. Subsequently, the effects of ARB on cell function and MAFLD in mice were studied, and 16S rRNA sequencing and metabolite analysis were conducted to explore the dual mechanism by which ARB exerts its effects through anti-apoptotic activity and reversal of gut microbiota imbalance.

## Materials and methods

### Establishment of the MAFLD animal model

Six-week-old male C57BL/6J mice (body weight, 16 ± 2 g) were purchased from the Experimental Animal Center of Shanxi Medical University (Production License number: SCXK (Jin) 2024-0004). Mice were housed in a barrier environment under a 12-h/12-h light/dark cycle with free access to food and water. ARB (purity, > 99.76%) was purchased from MedChemExpress (Monmouth Junction, NJ, USA, Cat No.: HY-N0192). And 60% fat-added feed was obtained from Dyets Company (Bethlehem, PA, USA, Cat No.: HF60). The animal model of MAFLD was established through high-fat diet (HFD) feeding. Based on our previous findings (Supplementary Table S1), two concentrations of ARB were selected to avoid liver damage in the experimental animals.

### Experimental design

After 1 week of adaptive feeding, mice were randomly divided into the following five groups (*n* = 6/group): normal control (Ctrl), HFD feeding (HFD), HFD feeding combined with 10 mg/kg/day ARB gavage (HFD + ARB-L), HFD feeding combined with 100 mg/kg/day ARB gavage (HFD + ARB-H), and HFD feeding combined with 5 mg/kg/day pioglitazone gavage (HFD + Pio) as a positive control (Liu et al. 2023). Simultaneously, the Ctrl and HFD groups received the same dose of solvent *via* gavage. The intraperitoneal glucose tolerance test (IPGTT) and insulin tolerance test (ITT) were conducted during weeks 8 and 9 of feeding, respectively. Body weight was recorded weekly to assess modeling efficacy. The entire experimental period lasted 12 weeks. After the experiment, the mice were anesthetized *via* an intraperitoneal injection of 120 mg/kg tribromoethanol (Meilunbio, China, Cat NO.: MB2548). Blood was collected from the medial canthal vein, and the serum was separated after centrifugation and stored at − 80 °C. Subsequently, laparotomy was performed to obtain a liver sample from each mouse. For fecal samples, three fresh fecal samples were taken from each mouse under aseptic manipulation and placed in 1.5-mL sterile Eppendorf tubes. Liver and fecal samples were immediately frozen in liquid nitrogen and then stored at − 80 °C. Under anesthesia, cervical dislocation was performed for euthanasia. All animal experiments complied with the Interpretation of AVMA Guidelines on Euthanasia of Animals: 2020 Edition. This work was reported according to the ARRIVE (Animal Research: Reporting In Vivo Experiments) guidelines (Kilkenny et al. 2010). The study was approved by the Ethics Committee of the Second Hospital of Shanxi Medical University (Ethics Number: DW2025016).

### Biochemical index detection

After centrifugation of blood samples, the supernatant is collected for biochemical index testing. Serum TC, TG (Jiancheng, Nanjing, China), LDL-C, and high-density lipoprotein cholesterol (HDL-C) levels were detected using commercial assays (Elabscience, Wuhan, China). FFA levels in the liver were detected using a commercial kit (Beyotime Biotechnology, Shanghai, China). Alanine aminotransferase (ALT) and aspartate aminotransferase (AST) levels were detected (Jiancheng, Nanjing, China) to evaluate liver function.

### Hematoxylin and eosin (HE) and oil red O staining

Samples were fixed in 4% paraformaldehyde for 24 h and dehydrated with gradient alcohol. Then, the samples were embedded in paraffin and cut into 4-µm sections for staining. Subsequently, xylene dewaxing was performed. After dehydration in gradient alcohol, HE staining was performed. For oil red O staining, fixed samples were dehydrated in gradient sucrose, embedded in OCT, and sliced into 10-µm frozen sections. Sections were immersed in oil red O staining solution for staining. After staining, images were obtained using a digital scanner (Pannoramic Scan, 3DHISTECH, Budapest, Hungary) for analysis. The NAFLD activity score (NAS) was used to describe the severity of liver lesions. NAS (0–8 points) specifically assesses steatosis (0 points, < 5%; 1 point, 5%–33%; 2 points, 33%–66%; 3 points, > 66%), ballooning (0 points, no balloon-like changes; 1 point, mild ballooning; 2 points, moderate-to-severe ballooning), and lobular inflammation (scored by the number of inflammatory foci per 200× field of view; 0 points, none; 1 point, 1–2; 2 points, 2–4; 3 points, > 4) as reported previously (Brunt et al. 2011).

### Establishing an in vitro model of MAFLD

This study used the mouse AML12 hepatocyte line (Procell, Wuhan, China). It was cultured in DMEM/F12 supplemented with 10% fetal bovine serum, 10 µg/mL insulin, 5 µg/mL transferrin, 5 ng/mL selenium, 40 ng/mL dexamethasone, and 1% penicillin/streptomycin (Yang et al. 2023). Oleic acid (OA) and palmitic acid (PA) (Kunchuang Biotechnology, Xian, China) were used to simulate the environment of lipid metabolism disorder. FFA working solution with a concentration of 0.5 mM was prepared by mixing OA with PA at a molar ratio of 2:1. To explore the effects of ARB on lipid deposition and cell viability of AML12 cells, oil red O staining and the Cell Counting Kit-8 (CCK-8) assay were used. Cells were treated with different concentrations of ARB for 24 h and stained with oil Red O staining solution (Solarbio, Beijing, China). Subsequently, the dye was extracted with isopropyl alcohol and transferred to a 96-well plate. The absorbance was measured at 500 nm. To evaluate cytotoxicity, different concentrations of ARB were added to the culture medium for 24 h, and cell viability was detected using the CCK-8 kit (NCM Biotech, Suzhou, China).

### Small interfering RNA (siRNA) construction and transfection

One day prior to transfection, cells were seeded into six-well plates to ensure 50%–70% confluency at the time of transfection. According to the instructions of the kit, transfection was performed using Lipo8000 (Beyotime Biotechnology, Shanghai, China) and after medium replacement, the cells were cultured for 48 h. STAT3 siRNA (si-STAT3) and negative-control siRNA (si-NC) were synthesized by HANBIO Corporation (Shanghai, China). The specific sequences of si-STAT3 were as follows: 5′-3′, CCUGAGUUGAAUUAUCAGCUU; and 3′-5′, GGACUCAACUUAAUAGUCGAA. The sequences of si-NC were as follows: 5′-3′, UUUUCUCCGAACGUGUCACGUTT; and 3′-5′, ACGUGACACGUUCGGAGAATT. Transfection efficiency was confirmed by detecting changes in STAT3 mRNA and protein expression *via* quantitative PCR (qPCR) and western blotting, respectively.

### qPCR

Total RNA was extracted from mouse liver tissue using TRIzol reagent (Beyotime Biotechnology, Shanghai, China), and its purity was determined using a Nanodrop spectrophotometer. cDNA was synthesized using a reverse transcription kit (Takara Bio, Dalian, China). The reaction conditions were 37℃ for 15 min and 85℃ for 5 s. Amplification was conducted using the LineGene 9600 Plus system (Bioer Technology, Hangzhou, China). The following reaction steps were used: pre-denaturation at 95 °C for 30 s, followed by 40 cycles of denaturation at 95 °C for 5 s, and annealing/extension at 60 °C for 34 s. β-actin were used as the internal control, and the cycle threshold (Ct) was recorded. The 2^−∆∆Ct^ value was calculated to evaluate the relative expression of mRNA. Table 1 lists the primer sequences.

**Table 1:** Primers used in the study

Gene	Forward primer (5′-3′)	Reverse primer (5′-3′)
β-actin	GGCTGTATTCCCCTCCATCG	CCAGTTGGTAACAATGCCATGT
BAX	TGCTGATGGCAACTTCAACTG	AAGTCCAGTGTCCAGCCCAT
BCL2	CTGAACCGGCATCTGCACAC	TGAGCAGCGTCTTCAGAGACA
Caspase 3	TGAAGGGGTCATTTATGGGACA	CCAGTCAGACTCCGGCAGTA
STAT3	TCAGCGAGAGCAGCAAAGAA	TACGGGGCAGCACTACCT

### SDS-PAGE and western blotting

Approximately 30 mg of mouse liver tissue were weighed, and protein was extracted using RIPA lysate (KeyGENBioTECH, Jiangsu, China) containing 1% protease and phosphatase inhibitors. The protein concentration was measured using the BCA method (Thermo Fisher Scientific, Waltham, MA, USA). SDS-PAGE was performed using the same amount of protein, and separated proteins were transferred to PVDF membranes (Thermo Fisher Scientific). After blocking at room temperature for 1 h, membranes were incubated overnight at 4 °C with the target antibodies, including β-actin (ABclonal, Wuhan, China), BAX (Cell Signaling Technology, Danvers, MA, USA), BCL2 (Cell Signaling Technology), caspase 3 (Cell Signaling Technology), cleaved caspase 3 (Zenbioscience, Chengdu, China), STAT3 (Proteintech, Wuhan, China) and P-STAT3 (Proteintech). The next day, the membranes were incubated at room temperature with goat anti-rabbit IgG-HRP (Cell Signaling Technology) for 1 h. The indicator marker (Yeasen, Shanghai, China) was placed in one lane for molecular weight indication. Images were collected using the ECL system (MiniChemi 610 plus, Beijing Sage Creation Science Co., Ltd., Beijing, China).

### Cellular thermal shift assay (CETSA)

CETSA was conducted according to a previously described method (Mao et al. 2025). Specifically, AML12 cells were divided into the phosphate-buffered saline (PBS) solvent control group and 100 µM ARB treatment group. After collection, cells were pre-incubated at 4 °C for 3 min, then heated at 42–62 °C for 5 min and subsequently cooled for 3 min. Freeze–thaw lysis using liquid nitrogen cycling was followed by centrifugation at 20,000 × g for 20 min at 4 °C. The supernatant, which contained soluble protein, was then collected. The expression of each target protein was detected by western blotting. The expression changes of each target protein were compared at different temperatures.

### 16S rRNA sequencing and analysis

Fecal samples (50 mg) stored at − 80℃ were placed in sterile centrifuge tubes. Then, 0.5 g of sterilized zirconium beads (0.1 mm in diameter) and 500 µL of sterile PBS buffer were added to each tube. After tightly closing their caps, the tubes were placed in a tissue grinder for mechanical disruption at a frequency of 30 Hz for 3 min. Then, the tubes were centrifuged at 200 × g for 5 min, and the sediment at the bottom of the tubes was discarded. Next, 200 µL of the supernatant were carefully aspirated from each tube and transferred to a new sterile centrifuge tube. According to the kit instructions, nucleic acid extraction of microorganisms from mouse feces was conducted using the OMEGA Soil DNA Kit (D5635-02, Omega Bio-Tek, Norcross, GA, USA). Primers 338 F (5′-ACTCCTACGGGAGGCAGCA-3′) and 806R (5 ′-GGACTACHVGGGTWTCTAAT-3′) were used to amplify the V3–V4 region of the bacterial 16S rRNA gene. Band size was detected by 1% agarose gel electrophoresis. The PCR products were purified using the Agencourt AMPure XP kit (Beckman Coulter, Inc., Brea, CA, USA) kit. Subsequently, the purified products were quantified using the Quant-IT PicoGreen dSDNA assay Kit (Thermo Fisher Scientific). Library construction of the samples was performed according to instructions of the NEbNext Ultra Library Prep Kit (New England Biolabs, Inc., Ipswich, MA, USA). High-throughput sequencing was performed using the Illumina NovaSeq 6000 platform (Illumina, Inc., San Diego, CA, USA) at BIOTREE Co., Ltd. (Shanghai, China). Then, the sequence and abundance table of amplicon sequence variants (ASVs) were obtained. Taxonomic annotations were performed using QIIME2 software (version 2024.2), and a confidence value greater than 0.8 was used as the annotation criterion. Bioinformatics analysis of the gut microbiota was conducted using the Biotree Lims2 and OmicStudio platforms.

### Fecal metabolomics analysis

Approximately 50 mg of each fecal sample were mixed with 500 µL of ice-cold methanol for homogenization. After settling, the mixtures were centrifuged at 4 °C and 20,000 × *g* for 10 min, and the supernatant was collected. Following the protocol provided by Lianchuan Bio (Hangzhou, China), the supernatant was analyzed by ultra-performance liquid chromatography–high-resolution mass spectrometry. Chromatographic separation was performed using an ACQUITY UPLC HSS T3 column (Thermo Fisher Scientific). Mass spectrometry was conducted using the Q Exactive™ Plus (Thermo Fisher Scientific) with dual-mode ion switching to comprehensively capture metabolites of varying polarities. The obtained data were analyzed using the OmicsStudio platform.

### Bioinformatics analysis

#### Data source

The transcriptome data of the Gene Expression Omnibus (GEO) database were retrieved using “Non-alcoholic fatty liver disease” as the keyword. Comprehensively considering its publication time and the size of the sample, GSE260666 was selected as the target dataset. This dataset used the GPL24676 Illumina NovaSeq 6000 (*Homo sapiens*) chip platform. Sixteen samples were included, including six healthy controls, six patients with MAFLD, and four patients with non-alcoholic steatohepatitis. The data of one sample numbered “GSM8122041” were not publicly available. Finally, samples from six healthy controls and five patients with MAFLD were used for subsequent analysis. R software (version 4.2.2) was applied to establish the correspondence between genes and probes and standardize the data. The limma package was subsequently used for differential expression analysis and volcano mapping. Before conducting gene set enrichment analysis (GSEA), the gene names were converted into Entrez IDs, and the NA values were deleted. Reference gene sets (C2: KEGG Medicus gene sets and H: HALLMARK_APOPTOSIS from the Molecular Signatures Database) were downloaded to evaluate the relevant pathways and molecular mechanisms. Statistical significance was denoted by *P* < 0.05 and false detection rate (FDR) < 0.25.

#### Network pharmacology and target prediction

The SMILE number of ARB was obtained by searching the PubChem database (https://pubchem.ncbi.nlm.nih.gov/) using the keyword “arbutin.” The 3D chemical structure of ARB was obtained using its SMILE model. Subsequently, the potential target point of ARB was predicted using the SwissTargetPrediction (http://www.swisstargetprediction.ch/), PharmMapper (https://www.lilab-ecust.cn/pharmmapper/index.html), and SuperPred databases (https://prediction.charite.de/) as previously reported (Daina et al. 2019; Gallo et al. 2022; Wang et al. 2016). The target UniProt IDs generated during this process was converted into the corresponding gene symbols using the UniProt database (https://www.uniprot.org/). MAFLD-related genes were validated from the GeneCards database (https://www.genecards.org/). Finally, the predicted targets of the three databases and the genes related to MAFLD were intersected, and the results were presented using a Venn diagram.

#### Gene ontology (GO) and Kyoto encyclopedia of genes and genomes (KEGG) enrichment analyses

DAVID (https://davidbioinformatics.nih.gov/) is a database containing abundant biological information and functional annotation (Dennis et al. 2003). The intersection genes shared by ARB targets and MAFLD were uploaded to the DAVID database. The potential biological functions and major signaling pathways were obtained using GO terms and KEGG pathways. The results were visualized using R software.

#### Protein–protein interaction (PPI) network construction and hub gene identification

The intersecting genes were further uploaded to the STRING database (https://cn.string-db.org/), an authoritative tool for analyzing protein interactions (Szklarczyk et al. 2023). The threshold of the minimum interaction score was set to 0.4 to obtain a file of connections between nodes. Subsequently, Cytoscape software (version 3.10.0) was used to visualize the PPI network. Using the cytoHubba plugin, the Matthews correlation coefficient (MCC) algorithm was applied to obtain the top 10 hub genes with the highest network connectivity for subsequent analysis.

#### Molecular docking and interaction analysis

Molecular docking and interaction analysis were used to analyze the interactions between ARB and hub genes. The protein structures of the hub genes were screened from the RCSB protein (https://www.rcsb.org/) and UniProt databases. The 3D structure of ARB was obtained from PubChem (https://pubchem.ncbi.nlm.nih.gov/). Prior to molecular docking, preprocessing of the target protein was performed using PyMOL software (version 3.1). Subsequently, molecular docking was performed using CB-DOCK2 with ARB as the ligand and the target protein as the receptor (Liu et al. 2022). PyMOL software (version 3.1) was used to visualize the interaction molecules and binding forces between ligands and proteins. Discovery Studio (version 4.5) was applied to further present the 2D forms of the docking results.

### Statistical analysis

Student’s t-test was used for comparisons between two groups. For comparisons among three groups, one-way ANOVA was used for data conforming to a normal distribution and homogeneity of variance. Otherwise, the Kruskal–Wallis H test was used. Statistical analysis was performed using IBM SPSS (IBM, Armonk, NY, USA) and GraphPad Prism (GraphPad, Boston, MA, USA). *P* < 0.05 denoted statistical significance. The 16S rRNA sequencing data were subjected to quality control and noise reduction using QIIME2 software. Species-level difference analysis was conducted using the Kruskal–Wallis H test, and multiple test corrections were performed using the Benjamini–Hochberg method. Further, linear discriminant analysis (LDA) effect size (LEfSe) was conducted to explore biomarkers with diagnostic value, and LDA score > 3 was set as the screening criterion. The non-targeted metabolomics sequencing data were preprocessed *via* missing value filling, normalization, and logarithmic transformation. Principal component analysis (PCA) and partial least squares discriminant analysis (PLS-DA) were used to analyze the differences in metabolic profiles between the groups. Differential metabolites were identified using the following criteria: variable importance in projection (VIP) ≥ 1.0 derived from PLS-DA, fold change (FC) ≥ 1.2 or ≤ 0.8, and Benjamini–Hochberg-corrected *P* < 0.05.

## Results

### ARB alleviated the pathological status in mice with MAFLD

**Fig. 1:**
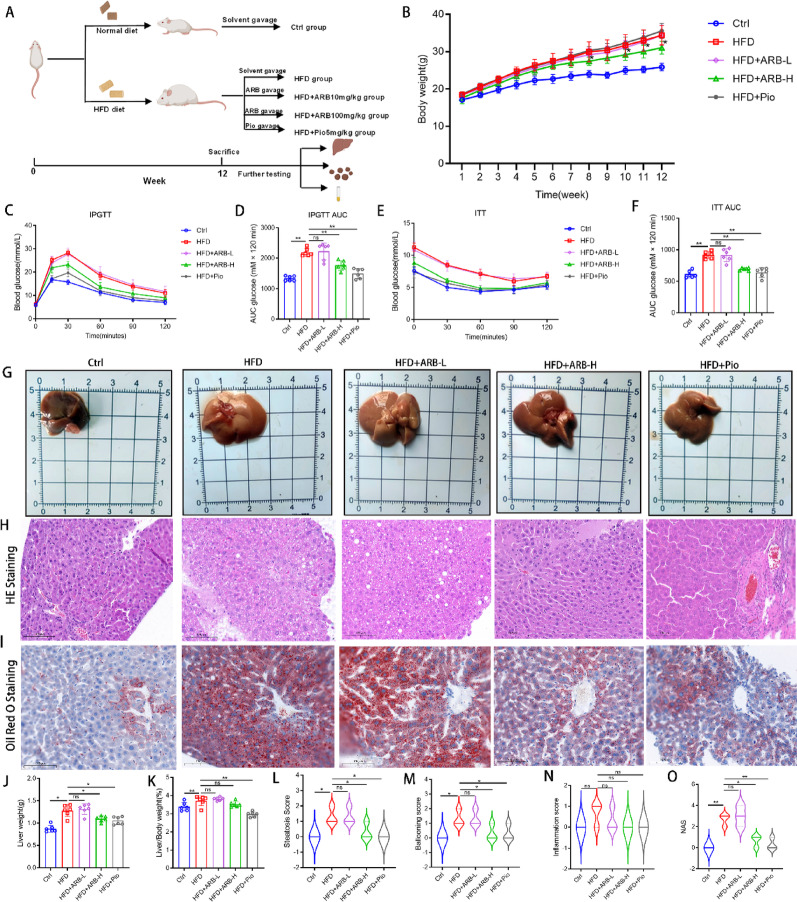
ARB administration affected liver histology in HFD-fed mice. **A** Schematic diagram of the 12-week animal intervention period for each group: Ctrl, HFD, HFD + ARB 10 mg/kg, HFD + ARB 100 mg/kg, and HFD + Pio 5 mg/kg. **B** Changes in mouse body weight in each group after 12 weeks of feeding. **C**,** D** The IPGTT was conducted during the eighth week of feeding to measure blood glucose changes and calculate the AUC. **E**,** F** The ITT was conducted during the ninth week of feeding to measure blood glucose changes and calculate the AUC. **G** Typical macroscopic morphology of the liver in each group of mice. **H** Liver HE staining (scale bar, 100 μm). **I** Liver oil red O staining (scale bar, 100 μm). **J**,** K** Changes in liver weight and liver/body weight ratio. **L–O** Pathological scoring of the liver based on HE and oil red O staining, including steatosis score, ballooning score, inflammation score, and NAS. All data are expressed as the mean ± standard error of the mean. * *P* < 0.05, ** *P* < 0.01. *ARB* arbutin, *AUC* area under the curve, *HE* hematoxylin and eosin, *IPGTT* intraperitoneal glucose tolerance test, *ITT* insulin tolerance test, *Pio* pioglitazone, *NAS* nonalcoholic fatty liver disease activity score, *ARB-L* 10 mg/kg ARB, *ARB-H* 100 mg/kg ARB, *Pio* pioglitazone, *Ctrl* normal control, *HFD* high-fat diet

At the end of the 12-week experiment, the HFD + ARB-H group exhibited significant weight loss compared with the findings in the HFD group, whereas the HFD + ARB-L group displayed no notable changes (Fig. 1B). Daily food intake was also statistically analyzed (Fig. S1A). The IPGTT results indicated that mice in the HFD group exhibited significant insulin resistance, and the area under the curve (AUC) was increased (*P* < 0.01). In the ARB-H intervention, insulin resistance was significantly alleviated, as indicated by the reduced AUC (*P* < 0.01, Fig. 1C, D). The ITT illustrated that insulin sensitivity was significantly reduced in the HFD group, as indicated by the higher AUC. These findings were significantly alleviated in the HFD + ARB-H group (*P* < 0.01, Fig. 1E, F). The effect was similar to that in the HFD + Pio group. However, no significant effect was noted in the HFD + ARB-L group (Fig. 1C–F). Fasting blood glucose levels at week 12 showed no significant difference among groups (*P* > 0.05, Fig. S1B). In contrast, both ARB-H and HFD + Pio treatment markedly reduced random blood glucose levels in HFD-fed mice (Fig. S1C).

Macro-morphological examination of the liver directly revealed hepatic injury. The liver volume was significantly increased in the HFD group, which was typified by elevated weight, a pale yellow surface, and rounded edges. These conditions were significantly improved in the HFD + ARB-H group, resulting in a lower liver weight and smaller liver/body weight ratio. No obvious changes were observed in the HFD + ARB-L group (Fig. 1G, J, K). In the HFD group, HE staining revealed marked ballooning degeneration within hepatocytes, with vacuoles of varying sizes containing lipid droplets. Minor inflammatory cell infiltration was observed within the lobules. Oil red O staining revealed extensive lipid droplets aggregated in brilliant red clusters. In the HFD + ARB-H group, ballooning degeneration, lipid droplet aggregation, and inflammatory infiltration were markedly reduced, in line with the effects of pioglitazone treatment. However, no significant improvement in liver injury was observed in the HFD + ARB-L group (Fig. 1H, I). Specific results were quantified using NAS (Fig. 1L-O). These results indicated that ARB effectively improved liver pathology in HFD-fed mice in a dose-dependent manner. ARB treatment at 100 mg/kg produced effects comparable to pioglitazone, whereas 10 mg/kg ARB exerted no significant effect.

### ARB improved blood lipid and liver function in HFD-fed mice

**Fig. 2:**
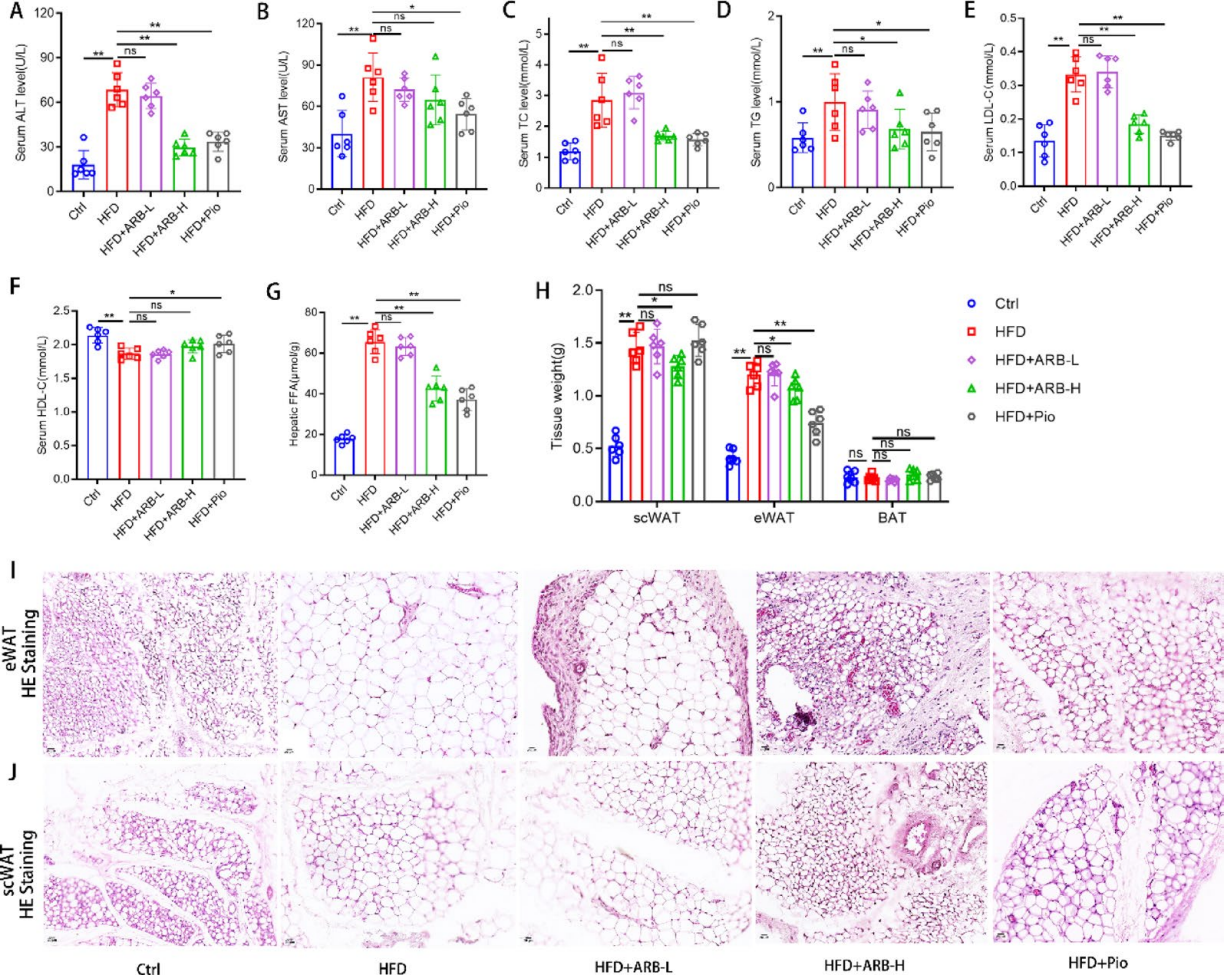
ARB treatment affected lipid levels and adipose histological staining in HFD-fed mice. **A**,** B** Serum ALT and AST levels in mice from each group. **C–F** Serum TC, TG, LDL-C, and HDL-C levels in mice from each group. **G** Liver FFA levels in mice from each group. **H** Statistical analysis of adipose tissue weight. **I** HE staining of eWAT (scale bar, 20 μm). **J** HE staining of scWAT (scale bar, 20 μm). All data are expressed as the mean ± standard error of the mean. * *P* < 0.05, ** *P* < 0.01. *ARB* arbutin, *ALT* alanine aminotransferase, *AST* aspartate aminotransferase, *HE* hematoxylin and eosin, *TC* total cholesterol, *TG* triglyceride, *FFA* free fatty acid, *HDL-C* high-density lipoprotein cholesterol, *LDL-C* low-density lipoprotein cholesterol, *eWAT* epididymal white adipose tissue, *scWAT* subcutaneous white adipose tissue, *BAT* brown adipose tissue, *ARB-L* 10 mg/kg ARB, *ARB-H* 100 mg/kg ARB, *Pio* pioglitazone, *Ctrl* normal control, *HFD* high-fat diet

Liver function and lipid levels were evaluated through biochemical tests. HFD-fed mice presented with severe liver function impairment, including elevated ALT and AST levels (both *P* < 0.01). ALT levels were reduced in the HFD + ARB-H group, whereas AST levels were not significantly changed (Fig. 2A, B). HFD-fed mice exhibited significantly elevated blood lipid levels, whereas serum TC, TG, and LDL-C levels were reduced in the HFD + ARB-H group, although HDL-C levels were not significantly changed. The findings in the HFD + ARB-H group were similar to those in the HFD + Pio group (Fig. 2C–F). However, blood lipid levels were not improved in the HFD + ARB-L group (*P* > 0.05, Fig. 2C–F). The weight of subcutaneous white adipose tissue (scWAT) and epididymal white adipose tissue (eWAT) was reduced in theHFD + ARB-H group compared with that in the HFD group (both *P* < 0.05), whereas no significant effect on brown adipose tissue (BAT) was observed (Fig. 2H). HE staining revealed extreme hypertrophy and heterogeneity in size regarding scWAT and eWAT in the HFD group. There were no significant changes in the HFD + ARB-L group. In the HFD + ARB-H group, adipocytes were smaller and more tightly packed. The effects were similar to those in the HFD + Pio group, although scWAT expansion was observed in this group (Fig. 2I, J). This suggested that high-dose ARB effectively alleviated lipid accumulation and improved liver function.

### ARB attenuated FFA-induced cellular lipid accumulation

**Fig. 3:**
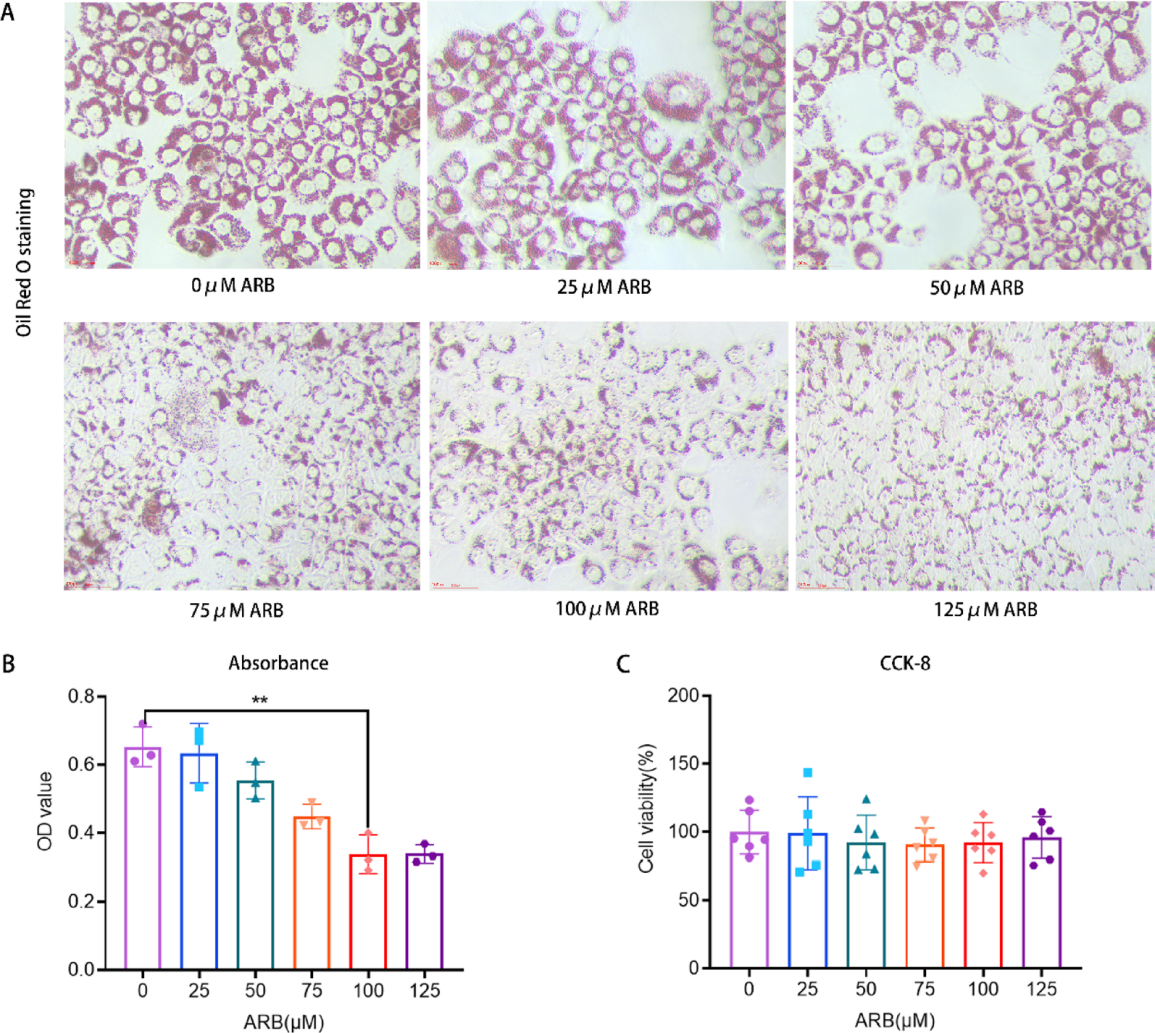
In AML12 cells, ARB suppressed lipid deposition in an in vitro MAFLD model induced by FFA exposure. **A** Oil red O staining revealing reduced lipid droplet aggregation at different ARB concentrations (magnification 200×). **B** The absorbance of the extracted oil red O staining solution was statistically analyzed. **C** Effects of different ARB concentrations on cell viability. All data are expressed as the mean ± standard error of the mean. * *P* < 0.05, ** *P* < 0.01. *ARB* arbutin, *FFA* free fatty acids, *CCK-8* Cell Counting Kit-8

To observe the effects of ARB on lipid accumulation in vitro, AML12 cells were used to simulate lipotoxicity with FFA exposure. The intervention was performed using ARB concentrations of 0, 25, 50, 75, 100, and 125 µM. The area stained with oil red O gradually decreased as the ARB concentration increased (Fig. 3A). The absorbance of the dye also gradually decreased after extraction with isopropanol (Fig. 3B). Lipid droplet formation was inhibited with equal efficacy by 100 and 125 µM ARB. CCK-8 was further used to evaluate cytotoxicity, and the results illustrated that ARB was not cytotoxic at any concentration (Fig. 3C). These findings indicated that ARB inhibited lipid aggregation in AML12 cells without affecting cell viability.

### RNA-Seq data acquisition for MAFLD samples

**Fig. 4:**
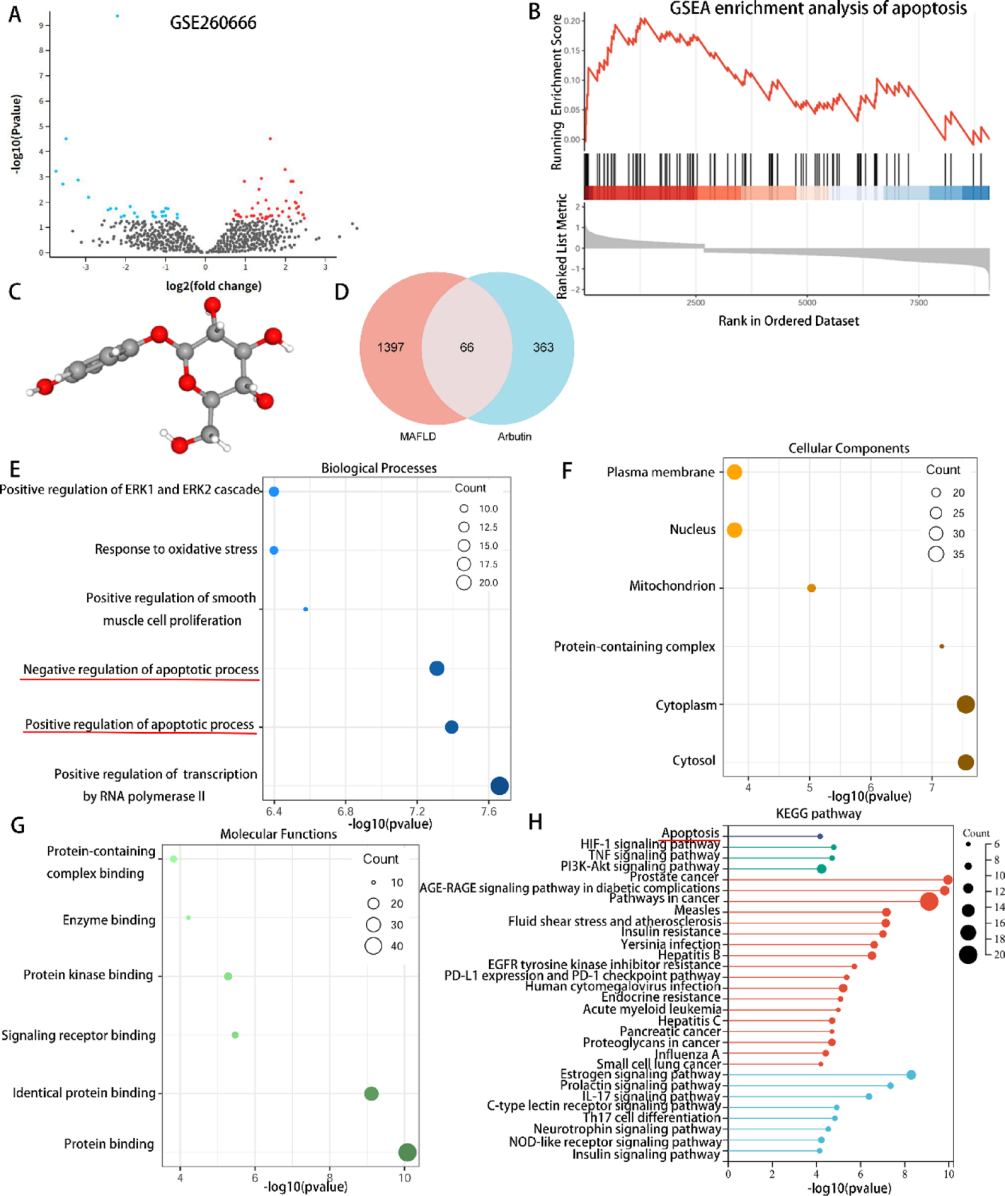
Bioinformatics analysis predicted the functional pathways of ARB. **A** Volcano plot of differentially expressed genes in the liver transcriptome. **B** GSEA based on the apoptosis pathway. **C** Three-dimensional structural schematic of ARB constructed using the PubChem database. **D** Venn diagram presenting the intersection of predicted ARB targets and MAFLD-related genes. **E–G** GO analysis of intersecting genes, including biological processes, cellular components, and molecular functions. **H** KEGG enriched pathways based on intersecting genes. *ARB* arbutin, *GSEA* gene set enrichment analysis, *GO* Gene Ontology, *KEGG* Kyoto Encyclopedia of Genes and Genomes

The transcriptome dataset GSE260666 was chosen to represent MAFLD samples, with |log2FC| > 1 and adjusted *P* < 0.05 as the screening criteria. Differential expression analysis revealed 539 upregulated genes and 380 downregulated genes, as presented in the volcano plot in Fig. 4A. GSEA based on apoptosis revealed that the apoptotic pathway was significantly activated in the disease group (*P* = 0.01, FDR = 0.08, Fig. 4B). These results suggested that apoptosis might be the key factor driving the development of MAFLD.

### Target prediction and GO and KEGG enrichment analyses

The SMILE number of ARB was obtained from the PubChem database to draw its 3D structure (Fig. 4C). Using the PharmMapper, SuperPred, and SwissTargetPrediction databases, 280, 90, and 78 ARB-related targets were obtained, respectively. Furthermore, 1463 potential pathogenic genes of MAFLD were obtained from the GeneCards database. The Venn diagram revealed that ARB and MAFLD have 66 overlapping target genes (Fig. 4D). GO and KEGG enrichment analyses were performed on these 66 overlapping genes. The enriched biological processes included positive regulation of transcription by RNA polymerase II and positive or negative regulation of apoptotic process (Fig. 4E). The enriched cellular components included cytosol, cytoplasm, and protein-containing complex (Fig. 4F). The enriched molecular functions included protein binding, identical protein binding, and signaling receptor binding (Fig. 4G). In addition, KEGG pathway analysis revealed that intersecting target genes might participate in apoptosis and inflammatory signaling (Fig. 4H). These findings suggested that the effect of ARB on MAFLD might be closely related to the regulation of apoptosis-related signaling pathways.

### PPI network construction and hub gene identification

**Fig. 5:**
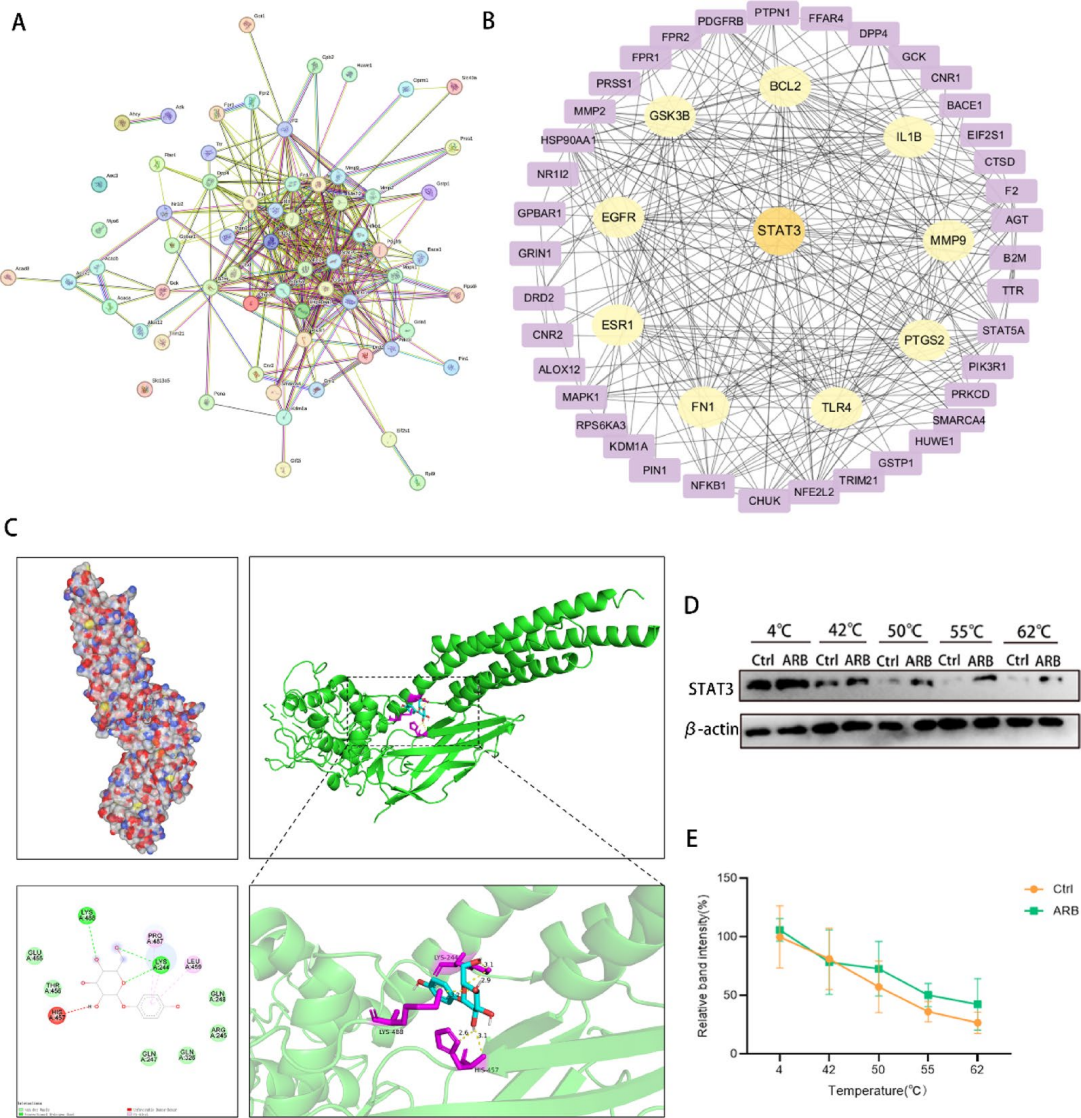
ARB target prediction and experimental validation. **A** Construction of a PPI network based on intersecting genes from the STRING database. **B** Identification of hub genes within the PPI network to predict the most relevant functional targets. **C** Molecular docking between ARB and STAT3. **D**,** E** CETSA confirming the interaction between ARB and STAT3. *ARB* arbutin, *STAT3* signal transducer and activator of transcription 3, *PPI* protein–protein interaction network, *CETSA* cellular thermal shift assay

PPI networks are important for revealing the interactions among proteins, biological processes, and functions. Based on the intersections of ARB-related targets and MAFLD screening, 66 genes were imported to the STRING database. Finally, 309 high-confidence interactions were identified (Fig. 5A). To highlight the importance of core genes for ARB intervention in MAFLD, the top 10 targets based on node connectivity were obtained by applying the MCC algorithm in Cytoscape software. The top 10 targets were STAT3, IL-1b, BCL2, matrix metalloproteinase 9 (MMP9), prostaglandin–endoperoxide synthase 2 (PTGS2), TLR4, fibronectin 1(FN1), estrogen receptor 1(ESR1), epidermal growth factor receptor, (EGFR) and glycogen synthase kinase 3 beta (GSK3B) (Fig. 5B). These 10 hub genes are believed to play important roles in the effects of ARB on MAFLD.

### STAT3 as a target of ARB

Combining the functions of the 10 target proteins, STAT3 was found to be closely related to the regulation of apoptosis. In a computer simulation, ARB was used as a ligand for molecular docking. The PDB file of STAT3 (PDB ID: 3CWG) was retrieved for molecular docking. ARB had a binding energy of − 7.1 kcal/mol with STAT3, and it formed hydrogen bonds with LYS244, LYS488 and HIS454 (Fig. 5C). Further analysis using CETSA demonstrated that ARB enhances the thermal stability of STAT3 as temperature increases (Fig. 5D). ARB treatment resulted in a clear rightward shift of the melting curve for STAT3 (Fig. 5E). These findings demonstrated that ARB forms stable complexes with STAT3 through hydrogen bond networks.

### ARB exerts anti-apoptotic effects in MAFLD

**Fig. 6:**
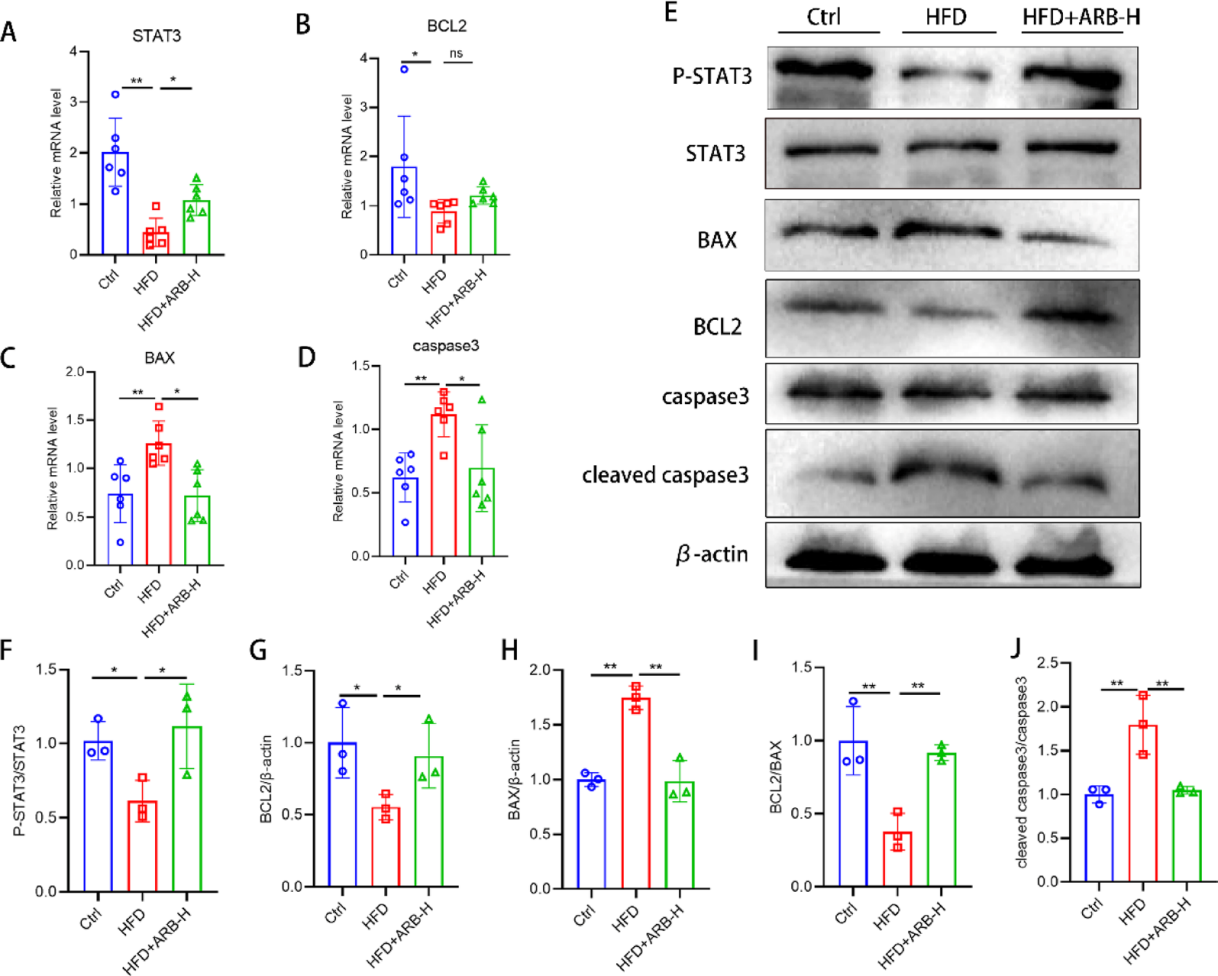
ARB exerted an anti-apoptotic effect on the livers of HFD-fed mice. **A–D** Relative mRNA expression of apoptosis-related genes in the liver. **E** Expression of apoptosis-related proteins. **F–J** Statistical analysis of the gray values of the bands for protein expression. All data are expressed as the mean ± standard error of the mean. * *P* < 0.05, ** *P* < 0.01. *ARB* arbutin, *ARB-H* 100 mg/kg ARB, *HFD* high-fat diet

qPCR and western blotting revealed that compared with the findings in the Ctrl group, the mRNA and protein expression of the pro-apoptotic factors BAX and caspase 3 was increased in the HFD group, whereas the expression of the anti-apoptotic factors STAT3 and BCL2 was decreased. These findings were significantly reversed in the HFD + ARB-H group (Fig. 6A–E). The results of protein quantification, as denoted by gray values (Fig. 6F–G). These were consistent with the results of bioinformatics analysis and further supported the critical role of apoptosis in MAFLD. The important role of ARB in regulating the apoptotic process by interfering with the STAT3–BCL2–BAX axis was also clarified.

### ARB reduced apoptosis *via* STAT3

**Fig. 7:**
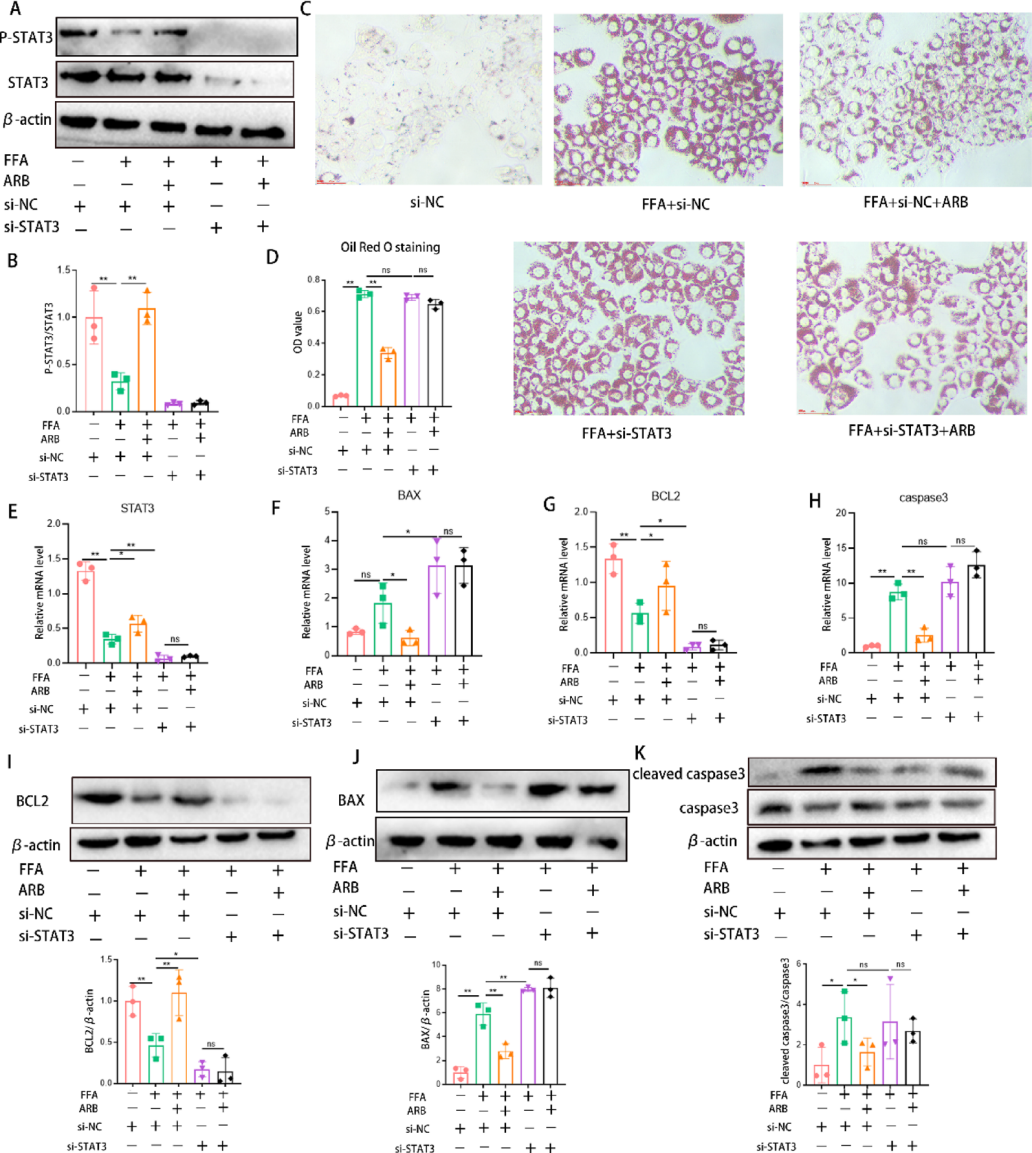
ARB exerted anti-apoptotic effects *via* STAT3. **A**,** B** Knockdown of *STAT3* in AML12 cells using siRNA. Knockdown efficiency was validated by western blotting, and band intensity values were quantified. **C** ARB intervention following *STAT3* knockdown in an FFA-induced in vitro MAFLD model. Oil red O staining was performed across different groups (magnification 200×). **D** Extracted oil red O staining solution was analyzed for absorbance. **E–H** The relative mRNA expression of apoptotic genes in different groups. **I–K** Detection of apoptosis-related protein expression following *STAT3* knockout. All data are expressed as the mean ± standard error of the mean. * *P* < 0.05, ** *P* < 0.01. *ARB* arbutin, *STAT3* signal transducer and activator of transcription 3, *FFA* free fatty acids

To validate STAT3 as a key target of ARB-mediated apoptosis regulation, we suppressed *STAT3* using siRNA, and the knockdown efficiency was shown in Fig. S1D, E. FFA and ARB were used simultaneously to intervene in the cells. The results suggested that the expression level of P-STAT3 was significantly up-regulated by ARB under FFA stimulation. After silencing *STAT3* with siRNA, the expression of P-STAT3 was significantly inhibited (Fig. 7A, B, E). Oil red O staining indicated that *STAT3* knockdown diminished the ability of ARB to reduce lipid accumulation in AML12 cells (Fig. 7C, D). Following *STAT3* knockdown, BCL2 expression was decreased, whereas BAX expression was increased. After *STAT3* knockdown, ARB no longer exerted anti-apoptotic effects (Fig. 7F–K). These findings confirmed that ARB exerts effects against MAFLD by regulating apoptosis through STAT3.

### ARB improved gut microbiota dysbiosis in mice with MAFLD

**Fig. 8:**
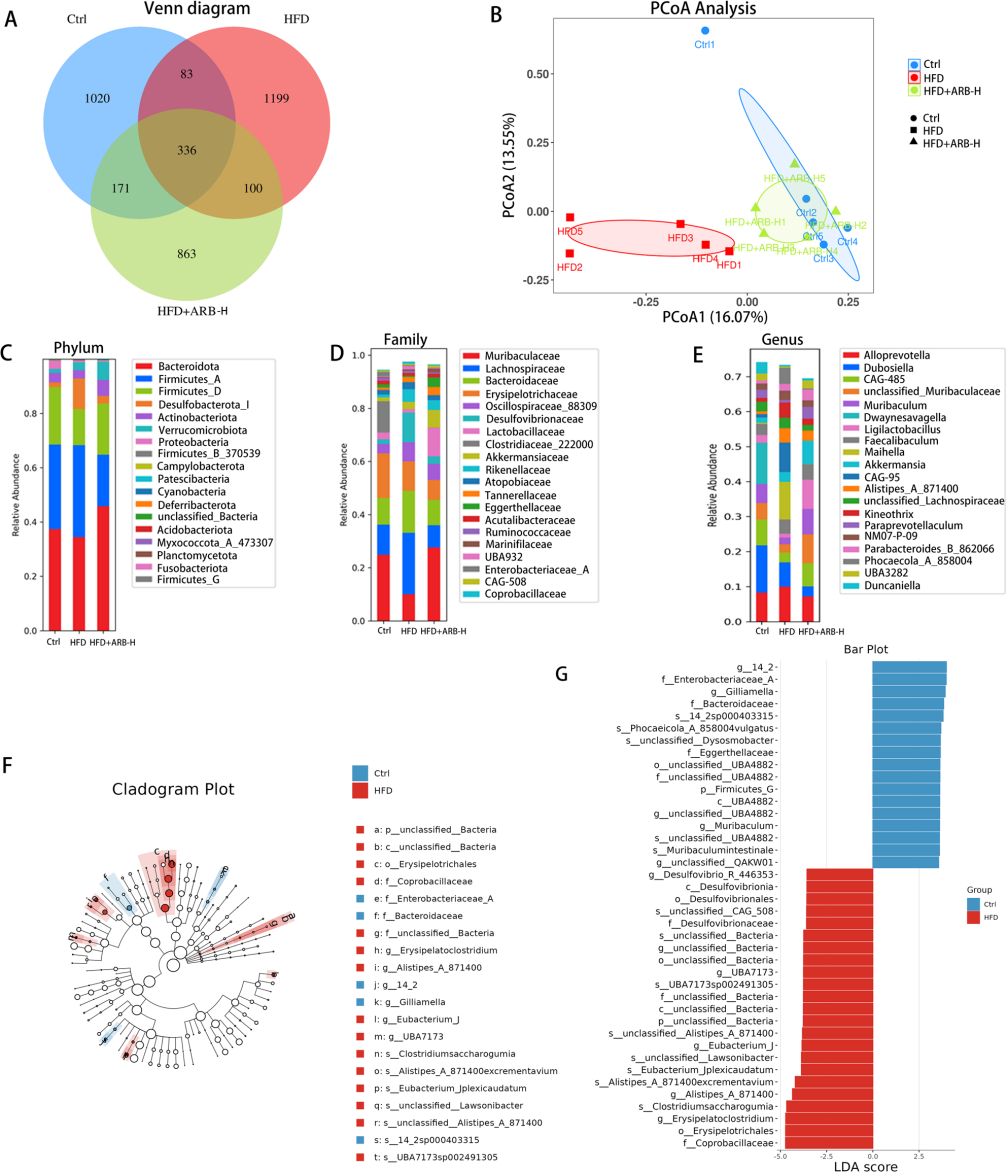
ARB altered the composition of the intestinal microbiota. **A** Venn diagram of the three groups of microbiota samples. **B** PCoA based on Bray–Curtis dissimilarity revealed distinct clustering of gut microbial communities among the three groups. PERMANOVA (999 permutations) revealed significant differences in microbial community structure between the groups: Ctrl vs. HFD: pseudo-F = 2.03, *P* = 0.023, q = 0.032; Ctrl vs. HFD + ARB: pseudo-F = 2.08, *P* = 0.009, q = 0.027; HFD vs. HFD + ARB: pseudo-F = 2.12, *P* = 0.032, q = 0.032. **C–E** Stacked bar plots present the relative STAT3, signal transducer and activator of transcription 3; abundance at the phylum, family, and genus levels. **F**,** G** Cladogram plot presenting taxonomic differences in the gut microbiota between the Ctrl and HFD groups and the linear discriminant analysis effect size bar plot displaying their significantly enriched taxa by the linear discriminant analysis score. *Ctrl* normal control, *ARB-H* 100 mg/kg ARB, *HFD* high-fat diet, *PCoA* principle coordinate analysis

**Fig. 9:**
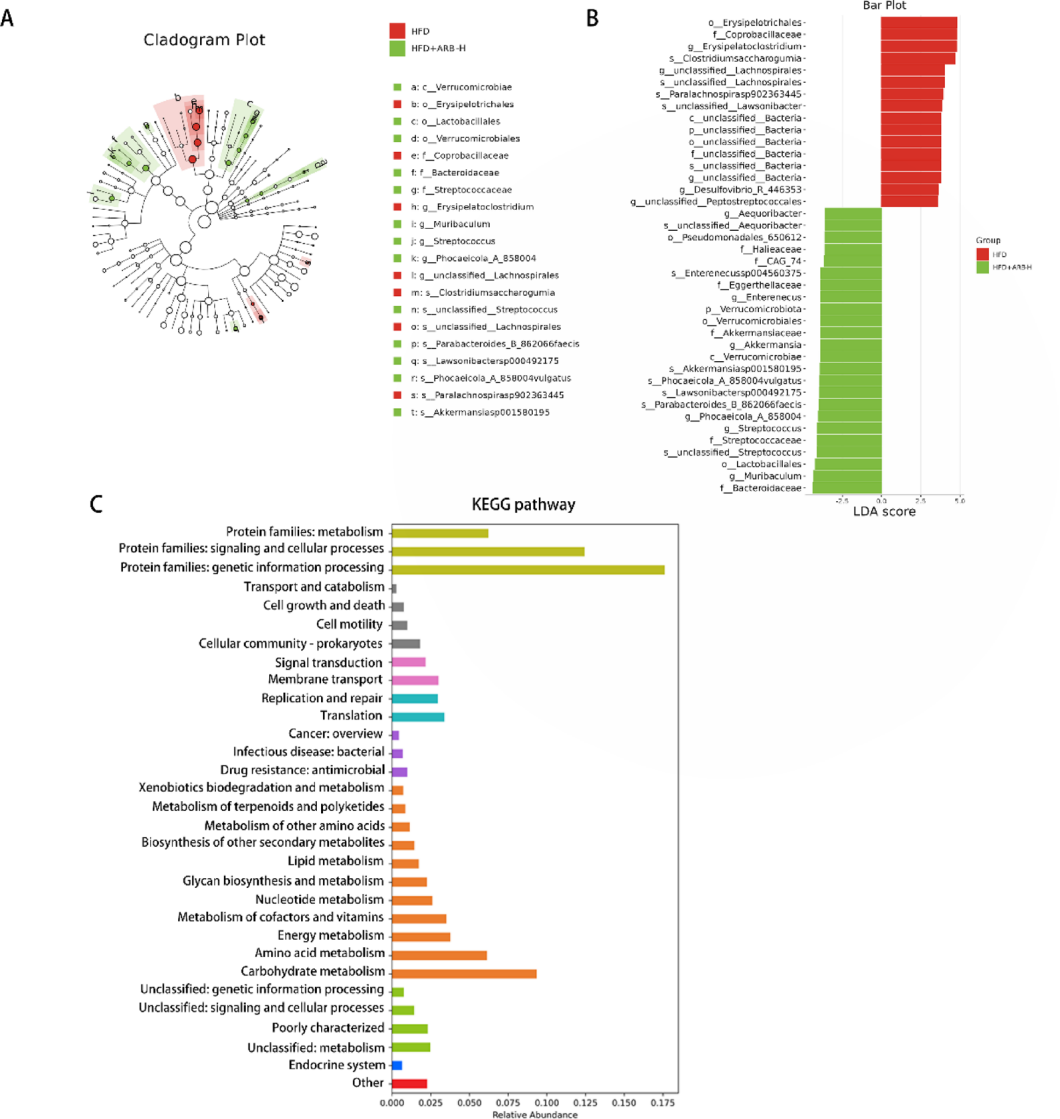
Functional prediction of differential gut microbes. **A**,** B** Cladogram plot presenting taxonomic differences in the gut microbiota between the HFD and HFD + ARB-H groups, and the LEfSe bar plot displaying their significantly enriched taxa by the LDA score. **C** KEGG pathway prediction for differential microbiota. *ARB* arbutin, *Ctrl* normal control, *HFD* high-fat diet, *ARB-H* 100 mg/kg ARB, *LEfSe* linear discriminant analysis effect size, *KEGG* Kyoto Encyclopedia of Genes and Genomes, *LDA* linear discriminant analysis

Considering the regulatory effect of ARB on the gut microbiota of mice with MAFLD as a potential mechanism, 16S rRNA sequencing was used to analyze the composition of the gut microbiota in each group of mice. The ASVs and shared ASVs across different groups were visualized using a Venn diagram (Fig. 8A). The β-diversity of the gut microbiota (assessed by Bray–Curtis dissimilarity) was visualized *via* principle coordinate analysis (PCoA) (Fig. 8B). PERMANOVA (999 permutations) confirmed significant differences in microbial community composition among all pairwise group comparisons: Ctrl vs. HFD (pseudo-F = 2.03, *P* = 0.023, q = 0.032), Ctrl vs. HFD + ARB-H (pseudo-F = 2.08, *P* = 0.009, q = 0.027), and HFD vs. HFD + ARB-H (pseudo-F = 2.12, *P* = 0.032, q = 0.032). At the phylum level, the abundance of Bacteroidota and Actinobacteriota was significantly decreased in mice with MAFLD, whereas that of Deslfobacterota_I was significantly increased. The aforementioned changes were reversed in the HFD + ARB-H group (Fig. 8C). At the Family level, the abundance of Muribaculaceae and Lactobacillaceae was decreased in the HFD group. However, that of Lachnospiraceae and Desulfovibrionaceae was obviously increased. These changes were reversed in the HFD + ARB-H group (Fig. 8D). At the genus level, the abundance of *Muribaculum*, *CAG-485*, and *Akkermansia* was decreased in the HFD group mice, and these changes were reversed in the HFD + ARB-H group (Fig. 8E). LEfSe revealed that, compared with the results in the Ctrl group, *Desulfovibrionia*, *Erysipelatoclostridium*, and Coprobacillaceae were enriched in the HFD group (Fig. 8F, G). High-dose ARB strongly influenced HFD-induced gut microbiota changes in mice. ARB-H administration increased the abundance of *Akkermansia*, *Muribaculum*, and Eggerthellaceae in mice (Fig. 9A, B). Overall, the gut microbiota of mice was significantly changed by HFD feeding and high-dose ARB treatment. This suggested a potential role for ARB in alleviating MAFLD *via* gut microbiota modification.

### Prediction of microbiota function and correlation analysis of inflammatory factors

Combined with ASV sequences and the KEGG database, PICRUST2 software was used to predict the function of different bacteria. The results indicated that the functions of microbial communities were centered on nutrient metabolism, including carbohydrate, energy, lipid, and amino acid metabolism. In addition, these pathways were involved in cell growth and death (Fig. 9C). Thus, the gut microbiota, *via* dysregulated metabolic functions, produced a large number of biologically active molecules, potentially driving lipid deposition in the liver through the gut–liver axis.

### ARB affected fecal metabolites in mice with MAFLD

**Fig. 10:**
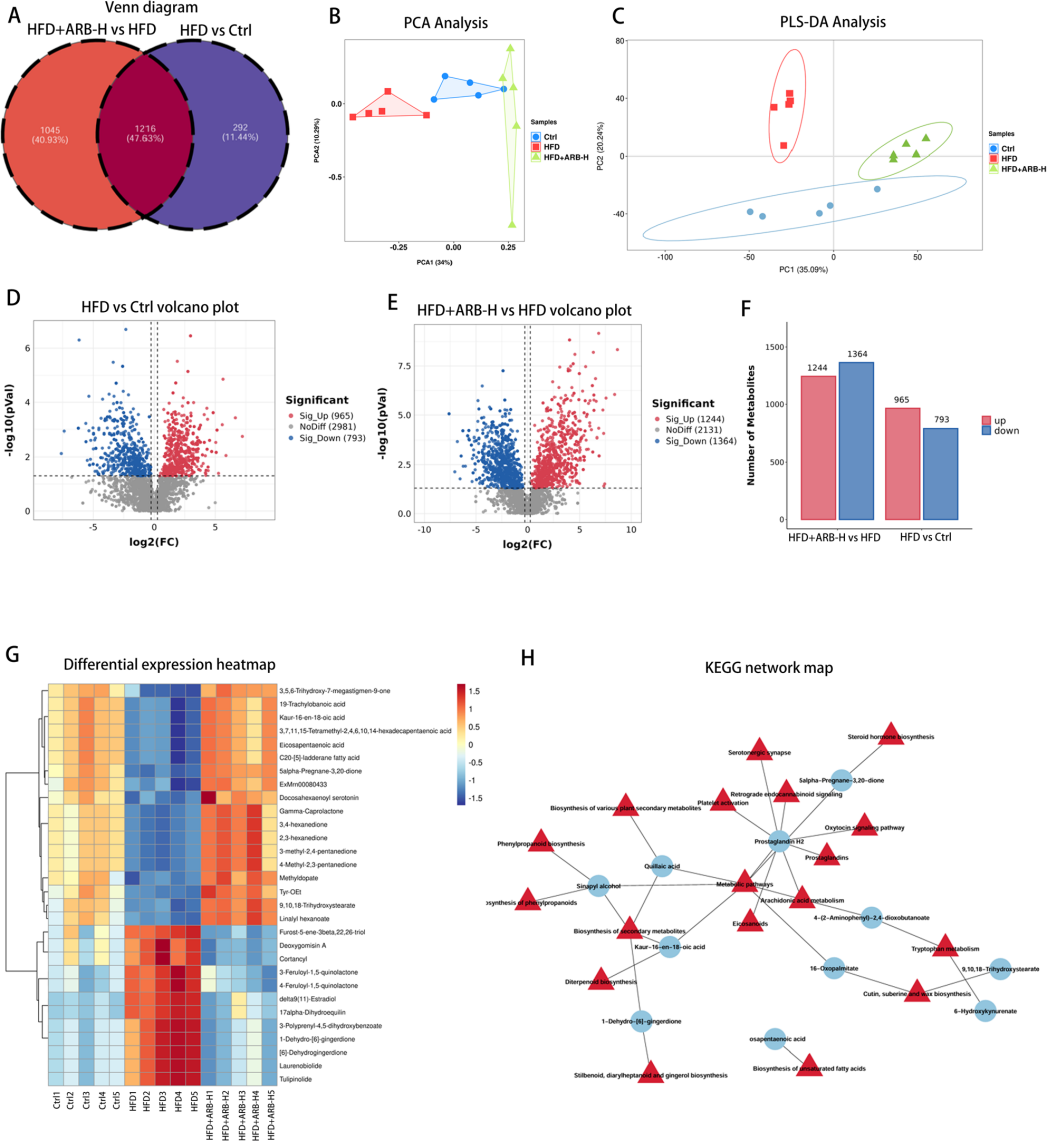
ARB altered fecal metabolites in mice with MAFLD. **A** Multi-group comparison of metabolites via the Venn diagram. **B** PCA of metabolites. **C** PLS-DA of metabolites. **D**,** E** Volcano plot of differential metabolites. **F** Statistics of differential metabolites based on the volcano plot. **G** Heatmap of differential metabolites in the Ctrl, HFD, and HFD + ARB-H groups. **H** KEGG enrichment signaling pathways of differential metabolites. *ARB* arbutin, *MAFLD* metabolic dysfunction-associated fatty liver disease, *PCA* principal component analysis, *PLS-DA* partial least squares discriminant analysis, *Ctrl* normal control, *ARB-H* 100 mg/kg ARB, *HFD* high-fat diet, *KEGG* Kyoto Encyclopedia of Genes and Genomes

Non-targeted metabolomics analysis was performed to evaluate the impact of ARB on fecal metabolites in mice with MAFLD. Venn diagram revealed 1216 common metabolites across the three groups (Fig. 10A). Compared with the findings in the Ctrl group, the HFD group featured 965 upregulated metabolites and 793 downregulated metabolites (Fig. 10D, F). After high-dose ARB intervention, 1244 metabolites were upregulated, and 1364 metabolites were downregulated (Fig. 10E, F). PCA and PLS-DA indicated significant differences in metabolite levels among the three groups (Fig. 10B, C). Cluster analysis demonstrated that ARB dramatically reduced the levels of pro-inflammatory mediators such as furost-5-ene-3β,22,26-triol, deoxygomisin A, and cortancyl. ARB elevated the levels of anti-inflammatory mediators including 9,10,18-Trihydroxystearate and eicosapentaenoic acid (EPA) (Fig. 10G). KEGG analysis revealed that differentially expressed metabolites were significantly enriched in pathways including lipid metabolism, bile acid metabolism, flavonoid biosynthesis, and isoflavone biosynthesis (Fig. 10H).

### Correlation analysis of MAFLD phenotype, gut microbes, and fecal metabolites

**Fig. 11:**
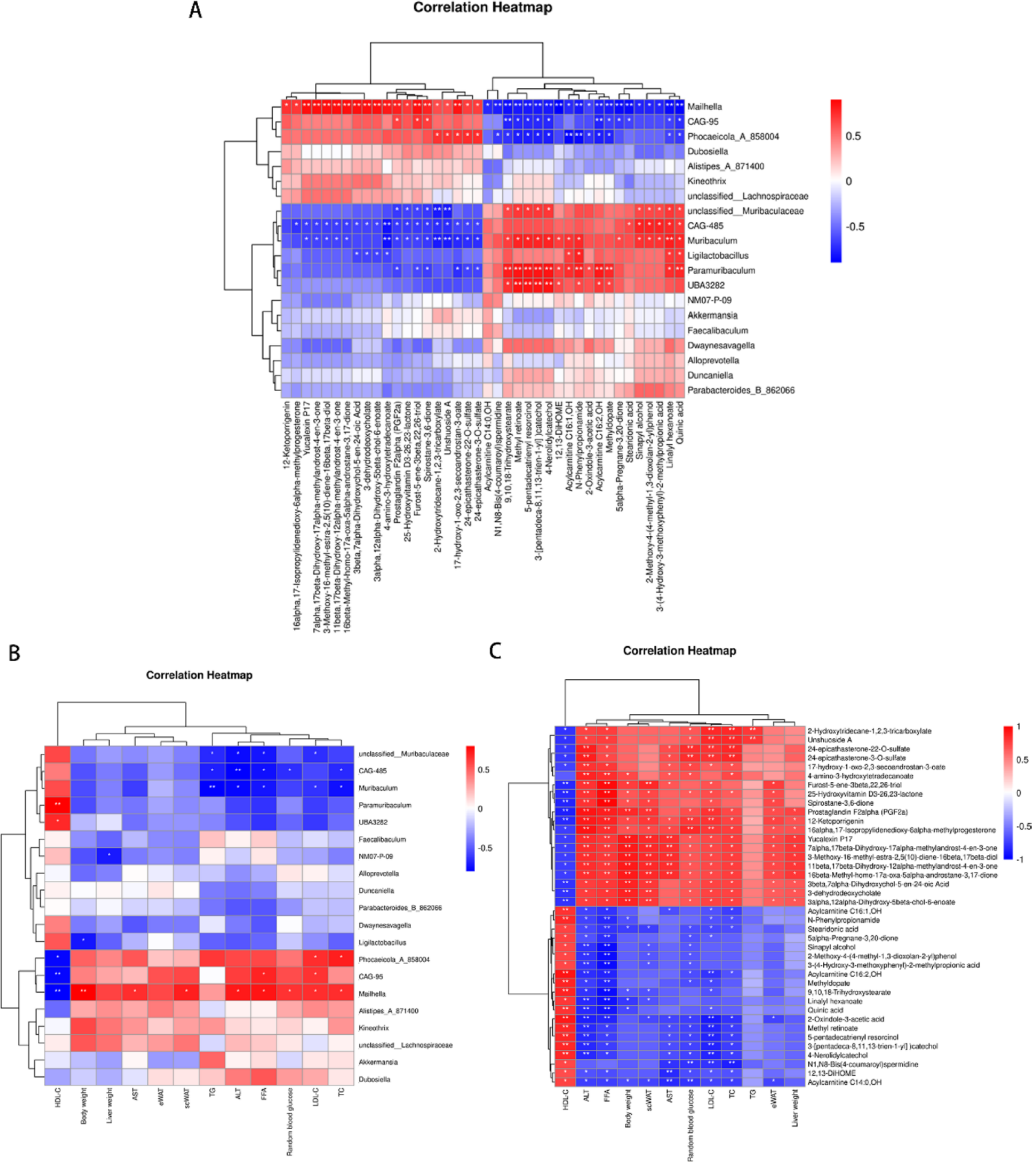
Spearman correlation analysis of fecal metabolites, gut microbes, and MAFLD phenotypes. **A** Correlation analysis between gut microbes and fecal metabolites. **B** Correlation analysis of gut microbes with hepatic biomarkers and biochemical indicators. **C** Correlation analysis of fecal metabolites with hepatic biomarkers and biochemical indicators. * *P* < 0.05, ** *P* < 0.01

Based on the current findings, we performed Spearman correlation analysis of metabolites, gut microbes, and biochemical indicators of MAFLD to elucidate potential associations. Correlation analysis between metabolites and gut microbiota revealed that increased abundance of *Ligilactobacillus*, *Muribaculum*, and *Paramuribaculum* was positively correlated with the upregulation of 20 metabolites after ARB treatment and negatively correlated with the downregulation of 20 metabolites after ARB treatment. Furthermore, decreases in the abundance of *Dubosiella* and *Mailhella* were negatively correlated with upregulated metabolites after ARB treatment and positively correlated with downregulated metabolites after ARB treatment (Fig. 11A).

Regarding MAFLD biomarkers, correlation analysis revealed that increases in the abundance of *Ligilactobacillus*, *Muribaculum*, and *Paramuribaculum* following ARB treatment were negatively correlated with liver weight, body weight, scWAT, eWAT, TC, TG, LDL-C, FFA, ALT, AST, and random blood glucose levels and positively correlated with HDL-C levels. Conversely, *Mailhella* exhibited opposite changes to those described for *Ligilactobacillus*, *Muribaculum*, and *Paramuribaculum* (Fig. 11B). Regarding metabolites, the 20 metabolites elevated after ARB treatment were negatively correlated with liver weight, body weight, scWAT, eWAT, TC, TG, LDL-C, FFA, ALT, AST, and random blood glucose levels and positively correlated with HDL-C levels. Conversely, the 20 metabolites decreased by ARB treatment displayed opposite correlations (Fig. 11C). These findings demonstrated that the gut microbiota plays a role in the effects of ARB against MAFLD by altering fecal metabolites levels.

## Discussion

MAFLD represents a major public health challenge globally. Its pathogenesis is complex, and treatment options remain insufficient. This study adopted the HFD-induced MAFLD mouse model as the research object to explore the intervention effect of the natural product ARB. We found that ARB could activate the anti-hepatocyte apoptosis pathway through STAT3. Meanwhile, ARB regulated the gut microbiota and fecal metabolites, restored gut microbiota homeostasis, and synergistically ameliorated the pathological manifestations of MAFLD. These findings provided some basis for the prevention and treatment of MAFLD.

Multiple studies have found that hepatocyte apoptosis is an important mechanism for the development of MAFLD and its progression to fibrosis (Dong et al. 2024; Li et al. 2020). In this study, we observed a decrease in the BCL2/BAX ratio in the liver and caspase 3 activation in mice with MAFLD, accompanied by a reduction in STAT3 expression. However, these changes were significantly reversed after ARB treatment. These results suggested that ARB had anti-hepatocyte apoptosis effects that might be related to the transcription factor STAT3. Further cell experiments confirmed that ARB could alleviate FFA-induced lipid deposition in hepatocytes. However, after *STAT3* knockdown, both the anti-apoptotic and lipid deposit-lowering effects of ARB were significantly reduced. Combining the discovery that ARB and STAT3 could stably bind, the findings collectively suggested that STAT3 is the core molecular target by which ARB exerts its protective effects.

It is worth noting that the role of STAT3 in MAFLD involved multiple pathways. This also explained the differences between the results of this study and some previous studies. Previous studies indicated that at specific stages of MAFLD progression (such as severe inflammation or fibrosis), STAT3 was abnormally activated in the liver because of the induction of systemic low-grade chronic inflammation (Park et al. 2023). This disfunction further drove the progression of MAFLD by regulating genes involved in glucose and liver cholesterol metabolism (Jiang et al. 2024). However, hepatocytes are under lipid stress in the early stage of MAFLD or simple steatosis, and STAT3 might function as a protective molecule. Min et al. found that STAT3 phosphorylation was almost undetectable in obese patients (Min et al. 2015). In another study, blueberry combined with probiotics inhibited BAX activation during the early steatosis stage of MAFLD by activating the STAT3 pathway, thereby alleviating liver steatosis (Zhu et al. 2018). This was similar to our finding that STAT3 downregulation in the early stage of MAFLD led to a decline in the anti-apoptotic ability of hepatocytes. ARB could upregulate STAT3, thereby inhibiting hepatocyte apoptosis and blocking the further development of MAFLD. This discovery enriches our understanding of the dual regulatory role of STAT3 in MAFLD and provides new ideas for the intervention of early MAFLD.

In addition to regulating the STAT3-mediated apoptosis pathway, this study confirmed that ARB could regulate the gut microbiota and fecal metabolites to construct a microecological environment conducive to improving MAFLD. This, in combination with the anti-apoptotic effect, ARB exerted dual effects in the treatment of MAFLD. Gut microbiota imbalance plays an important role in the occurrence and progression of MAFLD, as it can indirectly promote hepatocyte apoptosis by mediating chronic systemic inflammation and aggravating intestinal-derived injury (Meng et al. 2018). For instance, this study found that the relative abundance of Bacteroidota and Muribaculaceae was significantly reduced in mice with MAFLD, whereas ARB treatment could effectively reverse these changes. Among them, Bacteroidota is an important degrader of dietary fiber and potential producer of SCFAs (Sun et al. 2023). Species in this phylum exert anti-inflammatory effects and repair intestinal barrier function, thereby reducing the introduction of pro-apoptotic enterotoxin into the liver and alleviating hepatocellular injury. Muribaculaceae is an important family of sugar-degrading bacteria, and its depletion was closely related to metabolic disorders and intestinal barrier damage (Zhu et al. 2024). Therefore, ARB might repair the intestinal barrier by restoring the gut microbiota balance, thereby creating a systemic and local microenvironment for hepatocytes that reduces apoptotic stimulation.

Metabolism by intestinal microbes represents the core link that mediates microbial function. Fecal metabolites, as key mediators of interactions between microbes and the host, are key molecules affecting the fate of liver cells. Metabolomics analysis revealed that ARB could reshape the fecal metabolite profile of mice with MAFLD. Specifically, ARB significantly reduced the levels of pro-inflammatory mediators such as furost-5-ene-3β,22,26-triol, deoxygomisin A and cortancyl, thereby decreasing their effects on the apoptotic signaling pathway in hepatocytes. Meanwhile, ARB significantly increased the levels of metabolites such as 9,10,18-Trihydroxystearate and EPA. In particular, EPA is an ω-3 polyunsaturated fatty acid with anti-inflammatory and anti-apoptosis effects (Zou et al. 2023). Further correlation analysis illustrated that key microbes, such as *Ligilactobacillus*, *Muribaculum*, and *Paramuribaculum*, were positively correlated with the upregulation of anti-inflammatory metabolites and negatively correlated with the downregulation of pro-inflammatory and pro-apoptotic metabolites. This suggested that ARB could regulate the gut microbiota and fecal metabolites, thereby inhibiting the progression of MAFLD.

Certain limitations of this study must be acknowledged. Although this study clarified the association among ARB-mediated regulation of gut microbiota homeostasis, inhibition of hepatocyte apoptosis, and improvement of fatty liver, the causal link and synergistic regulatory network among these factors have not been fully elucidated. The dose–response relationship also remains to be clarified. Subsequent research should utilize functional experiments such as fecal microbiota transplantation and specific inhibition of apoptotic pathways. It is necessary to deeply explore the upstream and downstream pathways involved in the effects of ARB on the gut microbiota and apoptotic signaling pathways in the treatment of MAFLD.

## Conclusion

In summary, we revealed the mechanism by which ARB alleviates MAFLD through a multi-omics approach. This study found that ARB could slow the progression of MAFLD by regulating the structure of the gut microbial community and inhibiting hepatocyte apoptosis.

## Supplementary Information

Supplementary Material 1. Table S1

Supplementary Material 2. Fig. S1. **A** Record the dietary changes of mice in each group every week. **B, C** The fasting blood glucose and random blood glucose of mice in each group were detected at the end of the 12-week experiment. **D, E** Protein expression levels after STAT3 knockdown in AML12 cells.* P < 0.05, ** P < 0.01. *ARB* arbutin, *ARB-L* 10mg/kg ARB, *ARB-H* 100 mg/kg ARB, *Pio* pioglitazone, *Ctrl* normal control, *HFD* high-fat diet

## Data Availability

The public dataset used in this study was accessed through the GEO database. 16S rRNA gene sequencing data were stored in GeneBank database (https://www.ncbi.nlm.nih.gov/sra), registration number PRJNA1399090. Non-targeted metabolomics data were stored in the MetaboLights database (https://www.ebi.ac.uk/metabolights/), registration number MTBLS13626. Other data used to support the results of this study can be obtained from the authors as required.
