# Supplementary material for: Mechanism of arbutin in metabolic dysfunction-associated fatty liver disease based on multi-omics research
Source: Bioresour Bioprocess. 2026 Mar 25;13(1):36. doi: 10.1186/s40643-026-01032-5 (PMC13018519; doi:10.1186/s40643-026-01032-5)
Supplement: Supplementary file 1 — Supplementary Material 1. Table S1 [file 40643_2026_1032_MOESM1_ESM.docx]

| **Serum indicators** | **Ctrl** | **HFD** | **HFD+ARB**  **10mg/kg** | **HFD+ARB**  **100mg/kg** | **HFD+ARB**  **200mg/kg** |
| --- | --- | --- | --- | --- | --- |
| Serum TC level (mmol/L) | 0.82±1.2 | 3.02±0.46 | 3.11±0.55 | 1.93±0.47 | 1.08±0.21 |
| Serum TG level (mmol/L) | 0.46±0.06 | 1.07±0.18 | 1.08±0.21 | 0.67±0.94 | 0.73±0.19 |
| Serum ALT level (U/L) | 20.71±6.22 | 68.28±7.23 | 66.27±7.70 | 36.39±7.11 | 60.37±7.60 |
| Serum AST level (U/L) | 37.15±8.48 | 84.43±11.42 | 83.53±9.51 | 67.08±7.76 | 76.84±5.05 |

**Supplementary Table S1 Serum biochemical indicators in MAFLD models with different concentrations of ARB intervention**
