# Supplementary figures and images for: Mechanism of arbutin in metabolic dysfunction-associated fatty liver disease based on multi-omics research
Source: Bioresour Bioprocess. 2026 Mar 25;13(1):36. doi: 10.1186/s40643-026-01032-5 (PMC13018519; doi:10.1186/s40643-026-01032-5)

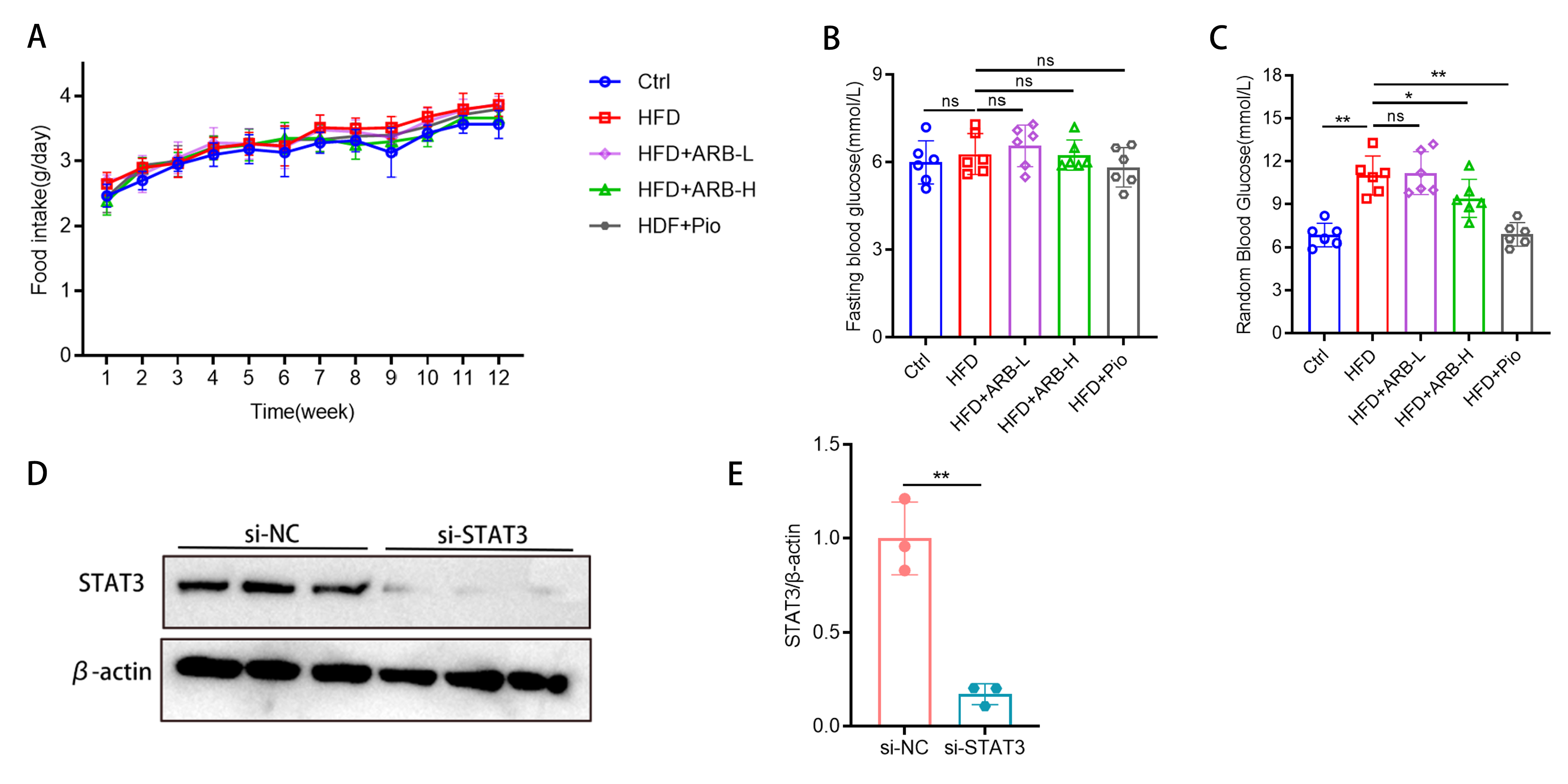

Supplement: Supplementary file 2 — Supplementary Material 2. Fig. S1. A Record the dietary changes of mice in each group every week. B, C The fasting blood glucose and random blood glucose of mice in each group were detected at the end of the 12-week experiment. D, E Protein expression levels after STAT3 knockdown in AML12 cells.* P < 0.05, ** P < 0.01. ARB arbutin, ARB-L 10mg/kg ARB, ARB-H 100 mg/kg ARB, Pio pioglitazone, Ctrl normal control, HFD high-fat diet [file 40643_2026_1032_MOESM2_ESM.png]
